# Effect of oestrogen therapy on faecal incontinence in postmenopausal women: a systematic review

**DOI:** 10.1007/s00192-020-04252-1

**Published:** 2020-03-04

**Authors:** Fiona L. Bach, B. Zeyah F. Sairally, Pallavi Latthe

**Affiliations:** 1 Birmingham Women’s Hospital, Mindelsohn Way, Birmingham, B15 2TG UK; 2 The University of Birmingham, Edgbaston Birmingham, B15 2TT UK

**Keywords:** Oestrogen therapy, Faecal incontinence, Postmenopausal, Systematic review, Hormone replacement therapy, Accidental bowel leakage

## Abstract

**Introduction and hypothesis:**

Faecal incontinence (FI) is prevalent in postmenopausal women. Oestrogen receptors have been identified in the anal sphincter and have been implicated in the pathogenesis and potential treatment. We sought to evaluate the literature regarding the impact of local and systemic oestrogen therapy on FI in postmenopausal women.

**Methods:**

A systematic review of all studies in postmenopausal women was performed to establish how oestrogen therapy affects FI. Eight articles were deemed eligible for inclusion following the Preferred Reporting Items for Systematic Reviews and Meta-Analyses (PRISMA) guidelines. Their quality was assessed using the Cochrane risk-of-bias tool (RoB-2) and Newcastle–Ottawa quality assessment scale.

**Results:**

One randomised controlled trial (RCT), two cohort studies, one observational and four cross-sectional studies were identified. The RCT showed an improvement in FI with anal oestrogen (*p* = 0.002), but this improvement was also observed in the placebo arm (*p* = 0.013) and no difference was seen between these groups. A prospective observational study demonstrated significant improvement with an oestrogen patch (*p* = 0.004), but had no control group. Conversely, a large prospective cohort study demonstrated an increased hazard ratio of FI with current (1.32; 95% CI, 1.20–1.45) and previous oestrogen use (1.26; 95% CI, 1.18–1.34) compared with non-users.

**Conclusion:**

All studies had a high risk of bias and had conflicting views on the effects of oestrogen on FI in postmenopausal women. This review has identified the need for further research in this area by highlighting the paucity of good research for evidence-based practice. We believe that a further RCT of local oestrogen is mandated to draw a valid conclusion.

## Introduction

Faecal incontinence (FI) in adults is a debilitating symptom that can severely impact quality of life (QoL) and represents a significant socioeconomic burden on the population [1]. It is likely to be underreported by patients owing to the social stigma associated with it. Variable definitions and the heterogeneous study populations make it difficult to establish the true incidence. A systematic review by Ng et al., which included 38 studies, found the prevalence to range from 2 to 21% for community-based adult females [2].

The pathogenesis of FI in postmenopausal women is likely to be multifactorial [3], but risk factors include injury of the obstetric anal sphincter (OASI) or the pudendal nerve during childbirth, increasing age [2] and pelvic floor changes secondary to menopause [4]. Oestrogen may be implicated and several studies have assessed the expression of sex hormone receptors in the bowel continence mechanism. Haadem et al., Oettling et al. and Parés et al. found oestrogen receptors in anal sphincter samples [5–7]. But in contrast, Rizk et al. did not identify oestrogen receptors in the rectal mucosa for either premenopausal or postmenopausal women [8]. Knudsen et al. found that female rats who had undergone a bilateral oophorectomy had a reduction in cross-sectional area of striated muscle of the anal sphincters compared with controls [9], suggesting a hormonal influence. It is known that lack of oestrogen after menopause contributes to the genitourinary symptoms of menopause, such as vaginal atrophy, urinary incontinence, recurrent urinary tract infections and dyspareunia and these have successfully been treated with local oestrogen therapy [10–13]. There is conflicting opinion as to whether oestrogen contributes to or protects from FI.

The objective of this review was to synthesise all the available evidence on the relationship between local and systemic oestrogen therapy and faecal incontinence in postmenopausal women.

## Materials and methods

We performed a systematic review of all studies in female humans without any language restrictions.

### Eligibility criteria

The population of interest was postmenopausal women, the intervention was the use of oestrogen therapy via any route, the comparator was no oestrogen use, placebo or other therapy and the outcome was faecal incontinence. All studies dealing with the above-mentioned population, intervention, comparator and outcome (PICO) of interest were evaluated.

### Search strategy and selection of studies

A literature search of the bibliographic databases including Ovid, Medline , Cochrane library, EMBASE, LILACS, Global Index Medicus and Cumulative Index to Nursing and Allied Health Literature (CINAHL) was conducted by a professional librarian. The search strategy was based on a number of relevant medical subheadings (MeSH), words and word variants. The following key words were used: “menopause” or “postmenopause” AND “oestrogen replacement therapy” or “HRT” or “oestrogen” or “hormone” and “replacement” and “therapy” or “oestrogen only” or “combined” and “oestrogen” and “progesterone” AND “faecal incontinence” or “faeces” or “bowel” or “anal” and “incontinence”.

The reference lists of the identified articles were searched and a grey literature search was performed to identify any other relevant articles or presentations (Annual Meetings screened for potential articles of interest: International Urogynecological Association [IUGA] 2014–2018 and International Continence Society [ICS] 2014–2017 [2018 not available]). Two reviewers then independently screened the titles and abstracts of all the articles and any disagreements were resolved through discussion or if required, by an independent third reviewer (PL). Full texts of the potentially eligible articles were then reviewed and assessed. One paper, only available in Portuguese, was translated into English for assessment.

The included studies were assessed for risk of bias using the Revised Cochrane risk-of-bias tool (RoB-2) [14] and the Newcastle–Ottawa quality assessment scale [15] where appropriate. The data were extracted independently by the two reviewers and entered onto a Microsoft Word table.

## Results

**Fig. 1:**
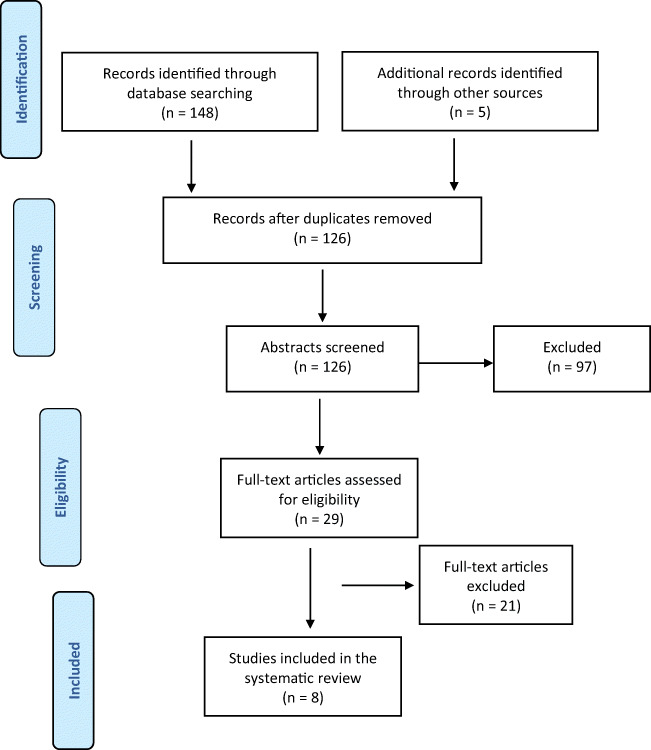
Preferred Reporting Items for Systematic Reviews and Meta-Analyses (PRISMA) 2009 flow diagram showing study selection process

A total of 148 articles were identified through the literature search. Twenty-nine full-text articles were retrieved and eight studies published between 1997 and 2017 were included in the final analysis. The selection of the articles has been reported in the Preferred Reporting Items for Systematic Reviews and Meta-Analyses (PRISMA) diagram (Fig. 1). The characteristics of the studies included are summarised in Table 1. There is 1 randomised controlled trial, 2 cohort studies, 1 observational and 4 cross-sectional studies. A meta-analysis could not be performed because of the heterogeneity of definition of the condition, intervention and the outcome, as well as the study designs.

**Table 1:** Summary of studies included in review

Reference, country	Population, sample size, age	Faecal incontinence definition	Methods	Control	Intervention	Outcome
[16], Dublin, Ireland	20 postmenopausal women (serum oestrogen <50 pg/ml) with established FI for mean 6.1 years—previously untreated. Average age 61 years (51–69); recruited from the gynaecology, menopause and coloproctology clinics at National Maternity and Mater Misericordiae Hospital	Continence grading score based on solid, liquid and flatal incontinence, soiling	Prospective observational study. Bowel function questionnaire, obstetric and menopausal history recorded. Anorectal physiology (anal manometry, anal endosonography and measurement of rectal sensation, anal electrosensitivity and pudendal nerve terminal motor latency) tested unblinded before and 6 months after HRT. Visual analogue score (VAS) for incontinence and daily and social activities	Nil	Oestrogen patch ± oral progestogen	90% -some improvements in symptoms with 25% completely symptom-free. Significant improvement in continence score (*p* = 0.0001), VAS social activity and daily activity (*p* = 0.001), mean resting pressure of the anal canal (*p* = 0.001) and mean squeeze pressure of the anal canal (*p* < 0.03)
[17], Chile	36 postmenopausal women with FI in the Colorectal Unit at Pontificia Universidad Católica de Chile. Average age 67 years (48–84) July 2005–2006	Wexner’s FI score >5	Prospective double-blind randomised trial. Endoanal USS to study anal anatomy and degree of continence by Wexner’s FI score (>5). QoL questionnaire (validated andaccepted for the spanish language) to evaluate impact	Topical placebo to the anal canal for 6 weeks	Topical estriol to the anal canal for 6 weeks	Both arms had statistically significant improvement in their Wexner FI scores (a difference of 50% considered successful). No statistical difference between groups (*p* = 0.521). The QoL questionnaire also showed minimal improvement for both groups
[4], USA	55,828 postmenopausal women from the Nurses’ Health Study, with no report of baseline FI. Mean age 73 years.	Report of at least one liquid or solid FI episode per month during 4 years of follow-up from self-administered biennial questionnaires	Prospective cohort study. Biennial questionnaire in 2008, 2010 and 2012 on lifestyle factors, medical diagnoses and hormone exposure (including menopausal hormone therapy (MHT)). “On average, how often in the past year have you experienced any amount of accidental bowel leakage?” Response categories included “never,” “less than 1/month,” “1–3/month,” “about once/week,” “several times/week,” or “nearly daily.” Women were considered to have FI if they reported incontinence of liquid or solid stool at least monthly	Postmenopausal women not on MHT with FI	Postmenopausal women on current MHT with FI and postmenopausal women previously on MHT with FI	Compared with women who never used MHT, multivariate hazard ratio for FI 1.26 (95% CI 1.18–1.34) for past MHT users and 1.32 (95% CI 1.20–1.45) for current MHT users. Risk of FI increased with longer duration of MHT use (*p* trend 0.001) and decreased with time since discontinuation
[18], Denmark	Aarhus University Hospital. 1 January 1976 to 1 November 1991. Women with recognised complete OASI (exposed—125) vs women delivering before and after with no recognised OASI (non-exposed—238). Mean age 50.4 years. Postmenopausal at final f/u—52 exposed (42%) and 126 non-exposed (54%). Postmenopausal on HRT—14 exposed (27%) and 35 non-exposed (28%)	Uncontrolled passage of flatus, liquid or solid stool	Longitudinal cohort study following OASI. Postal questionnaire, Wexner continence grading scale, St. Mark incontinence score and QoL questionnaire (TH Rockwood questionnaire)	Postmenopausal women not on HRT	Postmenopausal women on HRT	Post-menopausal HRT trend towards protective effect against FI (RR 0.66, 95% CI 0.41–1.06, *p* 0.06). However, adjusted RR following multivariate analysis 0.71 (0.44–1.31)—not significant.
[19], SouthernCalifornia	Kaiser Permanente members in Southern California. April 2004 to January 2005. 4,103 women, aged 25–84. 914 menopausal with no hormones (22.3%), 1,210 menopausal with past hormones (29.5%), 527 menopausal with current hormones (12.8%),	Anal incontinence included flatal, solid and/or liquid incontinence	EPIQ. Retrospective cross-sectional study random sampling within four age strata.	Menopausal with no hormones	Menopausal with previous and current hormones	877 cases anal incontinence/3,587. Compared with premenopausal (15.6%) Adjusted OR: menopausal, no HRT (22.1%) 1.22 (0.86–1.72); menopausal, past HRT (32.1%) 1.91 (1.35–2.71); menopausal, current HRT (30.8%) 1.65 (1.13–2.40)
[20], Alabama	November 1999 to February 2001 participants from Medicare beneficiary list in five counties in West Central Alabama (500 women and 500 men). Mean age 75.3± 6.7 (65–106)	In the past year, have you had any loss of control of your bowels, even a small amount that stained the underwear?	Cross-sectional survey. Interview using structured questionnaire	Women on HRT and FI	Women on HRT with no FI	From univariate analysis, 55.2% (32) suffered from FI vs 40.2% (176) with no FI—*p* 0.03. No difference in prevalence of FI with regard to HRT usage
[21], Spain	Patients attending primary care. 332 completed questionnaires (66.9%). Mean age 60.8 years	Any recurring episodes of involuntary loss of stool or flatus in the last 4 weeks?	Cross-sectional observational multicentre prospective study. Systematic random sampling of 10 primary care medical practices (linguistic knowledge, >18 years old, attended GP). Standardised face-to-face interview. Severity of FI assessed by St. Mark’s (Vaisey) Incontinence score (0–24). Required 307 patients for power of study	FI + HRT	No FI + HRT	Data were available for 229 women regarding HRT and FI. 28/192 (14.4%) women without FI were taking HRT. 5/37 (13.5%) women with FI were taking HRT. No statistical difference found in HRT usage with regard to FI (*p* 0.884). Duration of HRT usage longer in FI group, but not statistically significant (*p* = 0.766)
[22], Spain	100 women attending Menopause Outpatient Clinic (March 2003–January 2004). Postmenopausal (minimum 12 months of amenorrhea) and >45 years. Excluded—colorectal or gynaecological cancer, ulcerative colitis, Crohn’s disease or early menopause	The presence of one or more episodes of loss of solid or liquid stools in the last 30 days	Cross-sectional study: questionnaire, physical examination. Severity of FI by St. Mark score	HRT	No HRT	The history of use and timing of HRT was not associated with FI

*FI* faecal incontinence, *OASI* obstetric anal sphincter injury, *HRT* hormone replacement therapy, *USS* ultrasound scan, *QoL* quality of life, *EPIQ* Epidemiology of Prolapse and Incontinence Questionnaire, *MHT* menopausal hormone therapy, *VAS* visual analogue scale, *OR* odds ratio

Donnelly et al. [16] performed a prospective observational study of 20 postmenopausal women in 1997 with established FI and a low serum oestradiol (<50 pg/ml). Four women who had previously had a hysterectomy received an oestrogen patch for 6 months and 16 women with an intact uterus received the combination of an oestrogen patch and oral progestogen (50 μg oestradiol per 24 h and norethisterone acetate 1 mg daily for 12 days per cycle (Estrapak; Ciba Geigy, Basel, Switzerland). Patients were assessed using a bowel function questionnaire (no name given), a continence grading score (adapted from Pescatori et al. [23]) and a visual analogue score (VAS; no reference given) for the impact of incontinence on daily and social activities. Anorectal physiology, which consisted of anal manometry, anal endosonography, measurement of rectal sensation, anal electrosensitivity and pudendal nerve terminal motor latency was also tested. After 6 months of the HRT, 5 out of 20 patients (25%) reported being asymptomatic and a further 13 out of 20 (65%) reported some improvement. The bowel function questionnaire revealed a significant reduction in the number of women suffering from flatal incontinence (15 women to 5 women, *p* = 0.004), faecal staining of the underwear (10 to 2, *p* = 0.02) and defaecation urgency (13 to 6, *p* = 0.05), whereas a non-significant decrease in incontinence related to liquid stool (12 to 6), solid stool (6 to 3) and difficulty with defaecation (5 to 2) was demonstrated. No change was reported in the consistency of stool, requirement of digital manipulation and evacuation history. The intervention led to a significant improvement in the median scores for VAS for social activity (median score: 6 to 2, *p* = 0.001), daily activity (6 to 2, *p* = 0.001) and the continence score (15 to 8, *p* = 0.001). There were significant objective improvements in mean resting anal canal pressure (33 to 40 mmHg, *p* = 0.001) and mean squeeze pressure (39 to 43 mmHg, *p* = 0.03) on anal manometry [16]. A significant increase in the maximum tolerated rectal volume is reported but not reflected in the numbers published in the paper (187 ml to 170 ml). No difference was seen in the anal canal electrosensitivity (upper 5.3 to 5.2, *p* = 0.9; lower 4.4 to 4.1 mA; *p* = 0.3) or pudendal nerve terminal motor latency (PNTML) (right 2.45 to 2.5 ms, *p* = 0.5; left 2.46 to 2.44, *p* = 0.8). Thirty percent of women included in the study were found to have a previously unreported anal sphincter defect, but there were no differences in outcomes between these subgroups.

Pinedo et al. [17] performed a double-blind randomised trial to evaluate the effectiveness of topical oestrogen compared with placebo applied to the mucosa of the anal canal. Thirty-six postmenopausal women without hormonal substitution, with a Wexner’s FI score [24] of >5 and < 50% damage of the external sphincter were given topical estriol (Ovestin) or placebo to be applied three times a day for 6 weeks to the mucosa of the anal canal. A Wexner score and a quality of life score (validated and accepted for the spanish language) [25, 26], which included style of life, conduct, depression and embarrassment were performed at the beginning and end of the study. A difference of 50% was considered successful as per previous research [27, 28] and this was used to calculate the requirement for 17 patients in each branch. Both branches had similar patient characteristics. Both groups saw a statistically significant improvement in Wexner scores (oestrogen 12 to 7: *p* = 0.002; placebo 12 to 9: *p* = 0.013), but there was no difference between the two groups (*p* = 0.521). Both groups saw minimal non-significant improvements in quality of life scores following the protocol. Five patients in the estriol group experienced pruritus ani, which did not require treatment. No other side effects or complications were reported.

Staller et al. [4] reported from The Nurses’ Health Study, which was a prospective cohort of 121,701 US female nurses initiated in 1976, where biennial self-administered questionnaires were completed since its conception. In 2008, questions about FI were included in the questionnaire, allowing its association with menopausal hormone therapy (MHT) in postmenopausal women to be studied. They stated that they asked about oral MHT. After exclusions (23,393 died, 13,587 were lost to follow-up, 15,830 had no FI information, 5,737 had no MHT information, 7,325 had baseline FI), Staller et al. reported on a cohort of 55,828 nurses. Women returned questionnaires in 2008, 2010 and 2012 about lifestyle factors, medical diagnosis, FI and exposure to MHT. Lifestyle factors and medical diagnoses considered were BMI, smoking, physical activity, parity, history of cholecystectomy, diabetes, hypertension and presence of neurological disease. The definition of FI was at least one solid or liquid faecal incontinence episode monthly. The number of months used, current use and type of MHT were recorded. Because of the assumed association with hormones, the analysis included measures that were surrogates for endogenous and exogenous oestrogen exposure, including parity, age at menopause, oral contraceptive use, ovulatory duration and type of menopause (surgical, radiation, natural). Of the 55,828 postmenopausal women eligible for the analysis, 6,834 developed FI (48% liquid, 40% solid and 12% solid and liquid). The multivariate-adjusted hazard ratios for incident FI were 1.32 (95% CI 1.20–1.45) for current users and 1.26 (95% CI 1.18–1.34) for past users of MHT compared with women who had never used MHT. The type of MHT altered the risk of FI in a subgroup analyses of current users: the multivariate hazard ratio was 1.37 (95% CI 1.10–1.71) for current users of combined formulations when compared with current users of oestrogen-only preparations. Women who were current users of MHT were younger at menopause, less likely to be obese or have diabetes and more likely to have had a surgically/radiation-induced menopause and to have used oral contraception. A longer duration of MHT increased the risk of FI with a multivariate adjusted risk of 1.22 (95% CI 1.13–1.31) for 1–5 years of use, 1.24 (95% CI 1.15–1.35) for 6–10 years of use, and 1.32 (95% CI 1.23–1.41) for >10 years’ use (*p*_linear trend_ < 0.0001). The risk appears to return to baseline after >2 years of discontinuation of MHT [4].

Lawrence et al. [19] performed a cross-sectional study of patients enrolled in a health care plan in California (Kaiser Permanente), using the Epidemiology of Prolapse and Incontinence Questionnaire (EPIQ) [29] to compare the presence of anal incontinence (AI) in postmenopausal women with different oral hormone therapy usage versus premenopausal women. Out of 4,103 women available for analysis, 914 were postmenopausal with no hormones (22.3%), 1,210 were postmenopausal women who had used hormones in the past (29.5%) and 527 were on hormone therapy (12.8%) at the time of the study. There were a total of 877 cases of AI—defined as leakage of gas as well as solid and liquid stool—and they found that hormone therapy in postmenopausal women increased the risk of AI. 15.6% of premenopausal women had AI compared with 22.1% who were menopausal with no hormone therapy (adjusted OR 1.22 [0.86–1.72]), 32.1% who had had hormone therapy in the past (1.91 [1.35–2.71]) and 30.8% who were currently on hormone therapy (1.65 [1.13–2.40]).

Goode et al. [20] performed a similar cross-sectional survey on Medicare beneficiaries (Alabama), asking “In the past year, have you had any loss of control of your bowels, even a small amount, that stained the underwear?” A univariate analysis showed that significantly more (*p* = 0.03) women with FI were using oestrogen replacement therapy (55.2%) than those who did not have FI (40.2%), but in a multivariate analysis, it was found to be non-significant (*p* value not recorded).

Soerensen et al. [18] performed a longitudinal prospective cohort study in women who had undergone primary OASI repair identified from the Danish National Registry compared with controls on either side of the birth register. A questionnaire was sent out in 1989, 1992 and 2007. FI was defined as uncontrolled passage of flatus, liquid or solid stool and was graded by severity using the Wexner continence grading scale [24], the St. Mark incontinence score [30] and a QoL questionnaire (TH Rockwood Questionnaire [26]). Postmenopausal oral hormone therapy was included to look at long-term risk in these patients and showed a trend towards some protective effect against FI (relative risk [RR] 0.66, 95% CI 0.41–1.06, *p* 0.06). The adjusted RR following multivariate analysis was not significant at 0.71 (95% CI 0.44–1.31).

De Oliveira et al. [22] performed a cross-sectional study of 100 postmenopausal women in 2003–2004 attending the Menopause Outpatient Clinic of the State University of Campinas (Campinas, Spain). The definition of FI was the presence of one or more episodes of loss of solid or liquid stool in the last 30 days and the St. Mark score was used to assess severity. No association between hormone therapy and FI was found.

Bohle et al. [21] performed a cross-sectional observational multicentre study where 322 patients, selected at random, were questioned while attending a primary care setting (Barcelona, Spain). FI was defined as the involuntary loss of flatus or liquid/solid stool occurring during the last 4 weeks. The severity of FI was assessed using the St. Mark (Vaizey) Incontinence score (0–24) [30]. Data were available for 229 postmenopausal women regarding hormone therapy and FI. Of these, 28 out of 192 (14.4%) women without FI were taking hormone therapy and 5 out of 37 (13.5%) women with FI were taking hormone therapy. No statistical difference was found in hormone therapy usage with regard to FI (*p* = 0.884).

Eight studies were included in this systematic review to assess the effect of exogenous oestrogen on FI in postmenopausal women. The studies indicated variable results, from some benefit to worsening of FI. All the studies included had a risk of bias and were not found to be of high quality on the Cochrane risk-of-bias tool and Newcastle–Ottawa scale (Tables 2, 3).

**Table 2:** Revised Cochrane risk-of-bias tool for randomised trials (RoB 2)

RCT	Bias from the randomisation process	Effect of assignment to intervention	Effect of adhering to intervention	Bias due to missing outcome data	Bias in measurement of the outcome	Bias in selection of reported result	Overall risk of bias
Pinedo et al. [17]	Low	Some concerns	Low	Low	Low	Low	Some concerns

**Table 3:** Newcastle–Ottawa Quality Assessment Scale for Cohort Studies

Study reference	Soerensen et al. [18]	Staller et al. [4]
Study design	Cohort	Prospective cohort
Representativeness of the exposed cohort	0	0
Selection of the non-exposed cohort	1	1
Ascertainment of exposure	0	0
Demonstration that the outcome of interest was not present at the start of the study	0	1
Comparability on the basis of the design or analysis	0	2
Assessment of outcome	0	0
Adequate follow-up period	1	0
Adequacy of follow-up	1	0
Overall	3 out of 9	4 out of 9

The first paper, published in 1997, was a small observational study with neither a comparison group nor blinding and therefore had a high risk of bias [16]. Objective and subjective approaches to patient outcomes were assessed and the testing was very thorough. The intervention was not uniform, as some patients received combined oestrogen and progesterone replacement, whereas some had oestrogen alone but no comments were made as to whether this altered the outcome. The authors reported that all women completed the study, but did not specifically comment on compliance to medications. No information was given on the two patients who did not improve. The RCT performed [17] demonstrated “some concerns” when assessed using the Cochrane risk-of-bias tool [14]. It was double-blinded and had a robust randomisation method; however, it did not follow the “intention-to-treat” analysis (Table 2) and there is no record of compliance to medication. The nurses cohort study [4] has large numbers and is prospective; however, the population is selective (nurses only) and there is a risk of bias owing to self-completion of the questionnaires. It controlled for confounders associated with FI and other exposure to oestrogen. Initially, it was stated that the hormone therapy recorded in this study consisted of oral preparations only, although the authors later stated that they could not be sure that there were not some that were wrongly classified and that some of the participants were using topical preparations. The study by Soerensen et al. was not designed to look specifically at hormone therapy and postmenopausal FI; thus, although useful information can be taken from it, the study design cannot be assessed on this basis [18]. The remaining studies were cross-sectional surveys with inherent bias and some were not designed to explore faecal incontinence as the main outcome [19–21].

## Discussion

We believe that this is currently the only review collating all the evidence available related to the impact of oestrogen on FI for postmenopausal women. The role of oestrogen therapy (vaginal, anal, systemic) in the treatment of FI in postmenopausal remains unclear because of limited high-quality data, but there is biological plausibility.

The mechanism suggested for the improvement reported in the first study by Donnelly et al. [16] was increased resting pressure of the internal sphincter due to the improved functionality related to oestrogen causing alteration of collagen and elastic content of the pelvic floor. The subjective beneficial effects observed may have been related to an actual improvement in symptoms, but could also have been due to improved general wellbeing owing to increased circulating oestrogen from systemic administration and it is difficult to separate these effects [16]. The next study [17] found that topical anal oestrogens improved symptoms of FI; however, the placebo group also improved, highlighting a complex pathophysiology for FI with potential psychological overlay altering the patients’ perception of the condition and therefore altering the subjective outcomes. In contrast to these first two studies, in 2017, the large prospective cohort study [4] gave a hypothesis of oestrogen-mediated loss of connective tissue from the internal sphincter and levator ani to explain the increased FI with current and previous oestrogen use. The increased effect seen with combination therapy is attributed to progestin, causing both increased oestrogen receptors on the anorectum and a direct effect on oestrogen at a nuclear level. It is difficult to establish, in this particular study, whether exogenous oestrogen increases the risk of FI or whether women taking exogenous oestrogen are those who are affected most by both menopausal symptoms and FI and may have lower natural oestrogen levels. Although all the studies discussed add value to this systematic review, Staller et al.’s finding of increased risk of FI with oestrogen use is potentially a key finding that should be investigated promptly [4]. Faecal incontinence could be an under-reported but considerable side effect of the widespread use of hormone therapy in postmenopausal women.

With the ageing population worldwide, FI is a growing problem that needs to be addressed [2]. There is no definitive answer that we can give as to whether oestrogen therapy is helpful or harmful with regard to FI in postmenopausal women, but this review has certainly indicated the scope for further research in this field. Based on biological plausibility and lack of current studies establishing the role of oestrogen therapy on FI, high-quality studies examining the impact of oestrogen on FI are warranted. Standard definitions would aid our field in interpreting the impact of interventions on FI.

In conclusion, this review has highlighted the paucity of good-quality evidence in this area. We would suggest that a further randomised controlled trial (RCT) might be required to draw a valid conclusion. However, the challenges to this would be how to define faecal incontinence, in particular, the frequency of incontinence that is appropriate, and how much of an improvement is classed as a “success”. Using any systemic hormone therapy is associated with risks and an RCT to assess the effect of systemic oestrogen on faecal incontinence may be technically and ethically difficult to design; vaginal oestrogen might be more appropriate.

## Abbreviations

AI: Anal incontinence
FI: Faecal incontinence
HRT: Hormone replacement therapy
MHT: Menopausal hormone therapy
OASI: Obstetric anal sphincter injury
PICO: Population, intervention, comparator and outcome
QoL: Quality of life
RCT: Randomised controlled trial
RR: Relative risk
